# Feasibility of the string test for tuberculosis diagnosis in children between 4 and 14 years old

**DOI:** 10.1186/s12879-018-3483-0

**Published:** 2018-11-15

**Authors:** Karla T. Tafur, Julia Coit, Segundo R. Leon, Cynthia Pinedo, Silvia S. Chiang, Carmen Contreras, Roger Calderon, Milagros J. Mendoza, Leonid Lecca, Molly F. Franke

**Affiliations:** 1 Socios En Salud Sucursal Perú, Av. Túpac Amaru 4480, Comas, Lima, Peru; 2 Department of Global Health and Social Medicine, Harvard Medical School, Boston, MA USA; 3 Department of Pediatrics, Alpert Medical School of Brown University, Providence, RI USA; 4 Center for International Health Research. Rhode Island Hospital, Providence, RI USA

**Keywords:** Pediatric, Peru, Tolerability, Feasibility, Gastric aspirate

## Abstract

**Background:**

The enteric string test can be used to obtain a specimen for microbiological confirmation of tuberculosis in children, but it is not widely used for this. The aim of this analysis to evaluate this approach in children with tuberculosis symptoms.

**Methods:**

We conducted a cross-sectional study to assess children’s ability to complete the test (feasibility), and self-reported pain (tolerability). We examined caregivers’ and children’s willingness to repeat the procedure (acceptability) and described the diagnostic yield of cultures for diagnostic tools. We stratified estimates by age and compared metrics to those derived for gastric aspirate (GA).

**Results:**

Among 148 children who attempted the string test, 34% successfully swallowed the capsule. Feasibility was higher among children aged 11–14 than in children 4–10 years (83% vs 22% respectively, *p* < 0.0001). The string test was better tolerated than GA in both age groups; however, guardians and older children reported higher rates of willingness to repeat GA than the string test (86% vs. 58% in children; 100% vs. 83% in guardians). In 9 children with a positive sputum culture, 6 had a positive string culture. The one children with a positive gastric aspirate culture also had a positive string culture.

**Conclusion:**

Although the string test was generally tolerable and accepted by children and caregivers; feasibility in young children was low. Reducing the capsule size may improve test success rates in younger children.

## Background

The World Health Organization (WHO) estimated that one million of the 10 million cases of tuberculosis (TB) worldwide in 2017 occurred in children < 15 years of age [1]. This is likely an underestimate given that TB is notoriously difficult to diagnose in children. The WHO guidelines for diagnosing TB in children indicate evaluation of the child’s TB contact history and exposure, a test of infection (tuberculin skin test, interferon gamma release assay), chest radiograph, and bacteriological confirmation by culture [2]. As noted by Chiang, et al. [3] there are serious limitations to each step in the diagnostic process. Bacteriologic confirmation of pulmonary TB is the most certain method for determining disease status, but this test depends on sputum, which is difficult to collect in young children who are often unable to expectorate spontaneously [3]. The alternative sample type for bacteriologic confirmation of *Mycobacterium tuberculosis* in children who cannot produce sputum is gastric aspirate; however, the procedure to obtain this sample is invasive and it is not broadly available in resource-constrained settings due to a limited number of health providers trained in the procedure and/or a lack adequate space within primary health facilities [4–9]. Furthermore, the paucibacillary nature of TB in children decreases the sensitivity of acid-fast smear microscopy, mycobacterial culture and nucleic acid amplification tests, all of which often are negative in children with clinically diagnosed TB disease [10, 11].

The enteric string test, typically used to diagnose intestinal parasites, consists of ingestion of a small, dissolvable gelatin capsule containing a string that absorbs stomach fluids [12]. Because the string could also absorb sputum that has been swallowed, it has been proposed as a technique for obtaining lower respiratory specimens for tuberculosis testing in children. Several studies in adults have found that string samples have a diagnostic yield that is roughly similar to induced sputum [13–15]. The handful of studies in children have reported that > 80% could swallow the capsule and complete the test [4, 16, 17]; though this rate may vary by the child’s age, with the youngest children least able to swallow the capsule [13]. Among children who complete the test, diagnostic yield is comparable to sputum or gastric aspirate [13, 18]. In spite of its perceived simplicity and promise, the string test has not been widely adopted.

To respond to the need for a noninvasive clinical sample for routine TB diagnosis that is easily collected from children, we examined the feasibility and diagnostic effectiveness of the string test in children with suspected pulmonary TB in Lima, Peru.

## Methods

### Study population

The study population consisted of children in Lima, Peru who were consecutively enrolled in a larger pediatric TB diagnostic study, which aimed to identify sample types other than sputum that could be used for pediatric TB diagnosis. Children were less than 15 years old, had a history of contact with an adult with pulmonary TB and met at least one of the following criteria for inclusion in pediatric TB diagnostic studies, as defined by an expert panel [19]: persistent, unremitting, and unexplained cough for > 2 weeks, unexplained weight loss, unexplained fever for > 1 week, or unexplained fatigue or lethargy. Based on prior studies that found that the string test performed well in children as young as four years [16], we requested a string specimen from children four years of age and older. This analysis included all children that attempted the string test between May 2015 and December 2016.

### Standard of care for pediatric TB diagnosis in Peru

All children received the standard of care for pediatric TB diagnosis as defined by Peru Ministry of Health (MoH) guidelines [20]. Children provided two lower respiratory specimens and underwent chest X-ray, a tuberculin skin test (TST) and a physical examination by a MoH pediatric pulmonologist. Sputum induction and gastric aspiration were performed when was necessary. To preserve the viability of *Mycobacterium tuberculosis* for culture, we neutralized gastric aspirate samples at the time of collection by adding a few drops of sodium bicarbonate until the pH reached to between 6.8 and 7.2. All collected samples were then transported using cold chain (2 to 8 °C) to the laboratory for acid-fast smear microscopy and TB culture.

### String test procedure

Children were asked to swallow a nylon string coiled inside of a dissolvable, weighted gelatin capsule. Following an approximately eight-hour fast, a trained study nurse attached the proximal end of the string to the child’s cheek with tape and placed the capsule on the back of the child’s tongue. The child was asked to swallow the capsule with a glass of water. The string remained in situ for up to four hours of intragastric downtime. Drinking water was permitted if children complained of a dry or scratchy throat. The study team provided children with coloring books, puzzles, and electronic tablets with games while they waited to avoid potential agitation of the string. A study nurse removed the string by holding the proximal end and pulling it out of the stomach with a gentle tug, ensuring it made no contact with the tongue or other external surfaces. Using sterile scissors, the string was cut 10 cm from the distal end of the string that had been inside the stomach. The cut section was placed in a phosphate buffered saline solution with a Ph among 6.8 to 7.2 and transported at room temperature to the study laboratory within four hours of collection. All collected samples were then transported using cold chain (2 to 8 °C) to the laboratory for TB culture.

The capsules used in the study were manufactured locally in Peru. Capsule size and string lengths were determined based on participant age, the reference height of children from anthropometric tables developed by the Peru MoH, and estimates of the distance from mouth to stomach developed by Beckstrand et al. [21]. Encapsulated strings were designed with the following specifications: 1.6 cm-long capsules containing 50 cm of coiled string for children < 7 years old, 1.8 cm-long capsules containing 60 cm of coiled string for children 8–10 years old, and 2.2 cm-long capsules containing 70 cm of coiled string for children > 10 years old. All capsules were 0.5 cm in diameter.

### Culture procedures

String, sputum, and gastric aspirate samples were centrifuged at 3000 rpm (rpm); the pellet was then decontaminated with NALC-NaOH and neutralized with phosphate buffer. We performed culture using BACTEC MGIT 960 (Becton Dickinson, Franklin Lakes, NJ) on each sample type, following the manufacturer’s recommendations [22].

### Data collection

We collected demographic, epidemiologic, laboratory and clinical data via interviews with children and caregivers and clinical chart abstraction. Clinical data included signs and symptoms of TB and results of TST, chest x-ray, and clinical evaluation for TB disease.

Through surveys administered to children and guardians, we evaluated acceptability and tolerability of the string and gastric aspirate procedures. These surveys were first implemented five months after the study began and, therefore, only children and guardians enrolling in or after November 2015 completed these surveys.

#### Feasibility

We assessed the feasibility of the string test by the percentage of children who successfully swallowed the string capsule. We classified a child as having had an unsuccessful test if s/he could not swallow the capsule or hold it down.

#### Acceptability

We assessed acceptability in two ways. First, we asked guardians and children who attempted the string test and/or gastric aspiration how comfortable they felt with the procedure while it was underway and once it was over. For gastric aspiration, this was assessed for the first sample collected. Then, following completion of each procedure, we asked children and guardians to report whether they would be willing to repeat it.

#### Tolerability

**Fig. 1:**
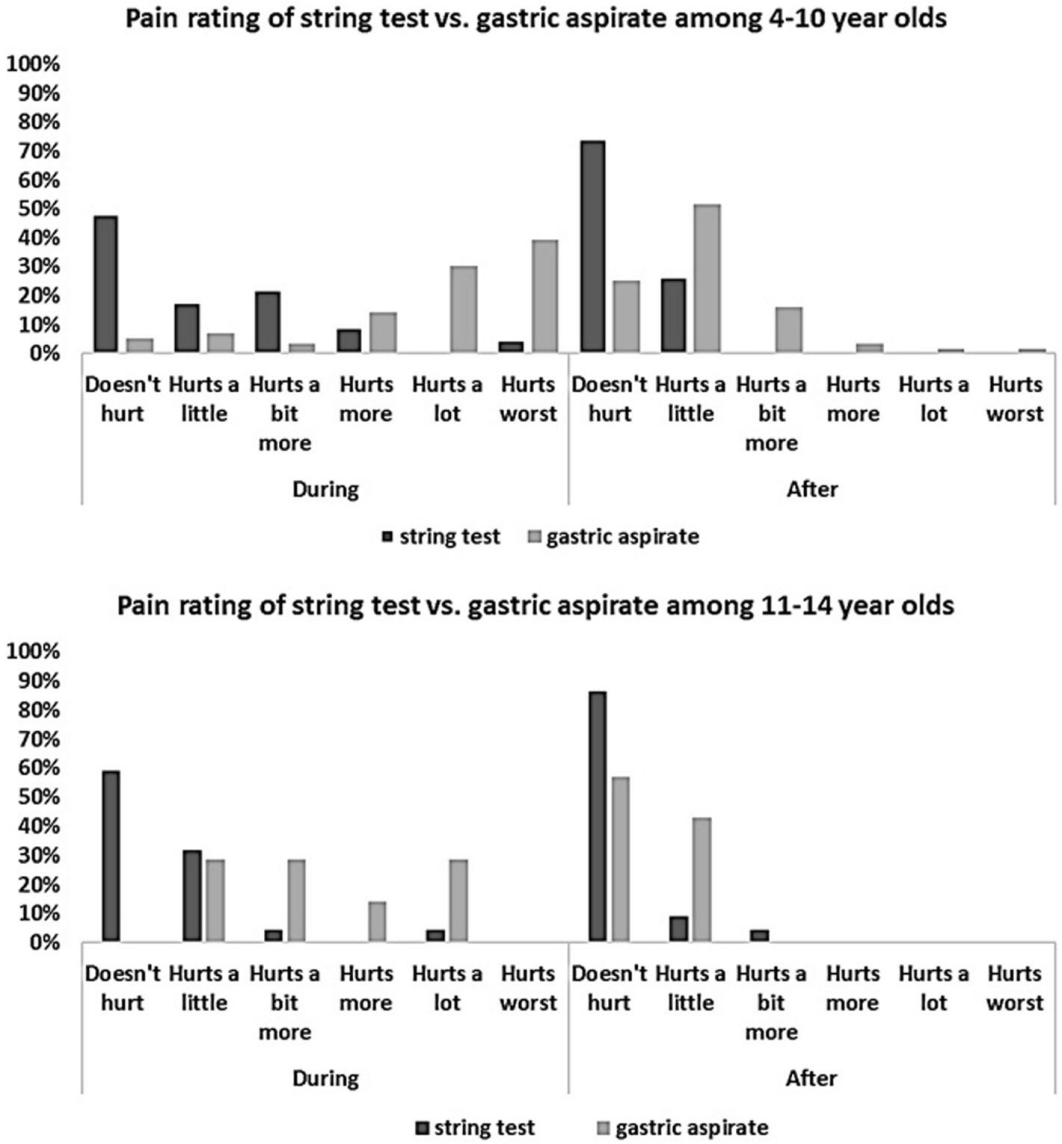
Self**-**reported pain levels by children during and after the string test and gastric aspiration, by age group

We evaluated tolerability among children who completed the string test using the Wong-Baker FACES® Pain Rating Scale [23, 24]. Children were asked to indicate with which of six cartoon facial expressions they most identified during and after the procedures. Response choices were ‘Doesn’t hurt’, ‘Hurts a little’, ‘Hurts a bit more’, ‘Hurts more’, ‘Hurts a lot’, and ‘Hurts worst’ (Fig. 1).

#### Diagnostic effectiveness

To assess diagnostic effectiveness, we compared culture results from string test samples to those from cultures conducted on gastric aspirate and sputum.

### Statistical methods

#### Feasibility

We calculated the percentage of children who successfully completed the string test among those who attempted it and compared success rates across age groups (4–10 and 11–14 year olds) using Chi-squared tests.

#### Acceptability

We compared the percentage of participants willing to repeat the string test and gastric aspiration using the Rao-Scott Chi-squared test. These methods accounts for repeated measures among participants who completed assessments for both sample types. We stratified percentages by participant type (child or adult) and child age group (4–10 or 11–14 years). Participants responding that they ‘Definitely agree,’ ‘Probably agree,’ or ‘Only agree if absolutely necessary’ to repeat the test were classified as ‘Willing.’ Those responding ‘Unlikely agree,’ ‘Don’t agree,’ or ‘Will not repeat procedure’ were classified as ‘Unwilling.’

We replicated these analyses for comfort level during and after the string and gastric aspirate procedures as reported by guardians. Responses of ‘Very comfortable,’ ‘Comfortable,’ or ‘Slightly comfortable,’ with a test were classified as ‘Comfortable.’ Responses of ‘Slightly uncomfortable,’ ‘Uncomfortable,’ or ‘Very uncomfortable’ were classified as ‘Uncomfortable.’

#### Tolerability

We describe the reported pain levels using Wong-Baker FACES® Pain Rating Scale [23] and graphed frequency of responses for the string and gastric aspirate procedures, stratifying responses by age group.

#### Diagnostic effectiveness

We reported the frequency of culture positivity for each sample type, stratified by age group.

Data were analyzed data using SPSS version 22.0 (IBM, Armonk, New York, USA) and Microsoft Excel™ (Microsoft Corporation, Seattle, WA).

## Results

We included 148 children (median age: 8.2 years, IQR: 5.5–10.0 years) in whom the string test was attempted. Table 1 provides descriptive statistics for the children and Table 2 shows an overview of study enrollment and procedures. Over half of children (54%) were under seven years old, and no child had known HIV-infection. Nearly all participants provided at least one sputum or gastric aspirate sample (*n* = 146, 99%) with 57% (*n* = 82) providing sputum (74 spontaneous and 8 induced samples, two children provided one of each), and 51% (*n* = 75) providing gastric aspirates. Thirty-two children were diagnosed with TB by a pediatric pulmonologist, of whom 12 (38%) had bacteriologic confirmation of TB by culture of sputum or gastric aspirate.

**Table 1:** Characteristics of 148 children (ages 4 to 14 years) who attempted the string test

Characteristics	4–10 years old *N* = 119 (80%)		11–14 years old *N* = 29 (20%)		TOTAL *N* = 148	
	*n*	%	n	%	n	%
Female sex	55	46	15	52	70	47
Clinical symptoms (last 4 weeks)						
Cough	117	98	28	97	145	98
Productive cough (among those with cough)*	88	74	19	66	107	72
Hemoptysis	5	4	3	10	8	5
Chest pain	33	28	10	34	43	29
Fever	37	31	11	38	48	32
Dyspnea	34	29	8	28	42	28
Loss of appetite	50	42	11	38	61	41
Night sweats	33	28	7	24	40	27
Vomiting	24	20	6	21	30	20
Sore or itchy throat	56	47	10	34	66	45
Pain with swallowing	26	22	6	21	32	22
Hoarseness	35	29	5	17	40	27
Positive tuberculin skin test (TST) (*n* = 133) **	53	48	14	61	67	45
Exposure to an adult with positive sputum smear test	109	92	26	90	135	91
Provided a spontaneous sputum sample	53†	45	21‡	72	74	50
Provided an induced sputum sample	5	4	3‡	10	9	6
Provided a gastric aspirate sample	68†	57	7	24	75	51
Result of evaluation for TB disease (*n* = 147) ẟ						
Clinically diagnosed TB without culture confirmation	19	16	1	3	20	14
Culture-confirmed TB	4	3	8	28	12	8
Not TB	95	81	20	69	115	78

*Percent with productive cough was calculated among those with cough

**Percentages for TST results were based on available results: *N* = 110 for 4–10 year olds, *N* = 23 for 11–14 year olds

†Seven children provided one spontaneous and one gastric aspirate sputum sample

‡Two children provided one spontaneous and one induced sputum sample

**Table 2:** Data collection for 148 children who attempted the string test (ages 4 to 14 years) and their guardians

	n/N	%
At least 1 upper respiratory sample collected	146/148	99%
Gastric aspirate	75/148	51%
Sputum (spontaneous and or induced)	82/148	55%
Successful string test	50/148	34%
Enrolled after survey implementation (November 2015)	124/148	84%
*String test attempted*		
Caregiver comfort after string test or attempt	111/124	90%
Caregiver willingness to repeat the string test	112/124	90%
Child willingness to repeat the test	112/124	90%
Children: Wong Baker FACES® scale after string test or attempt	114/124	92%
*String capsule swallowed*	50/124	40%
Caregiver comfort during procedure	43/50	86%
Children: Wong Baker FACES® scale during the procedure	45/50	90%
*Gastric aspirate collected*	63/75	84%
Caregiver comfort during procedure	63/63	100%
Caregiver comfort after procedure	63/63	100%
Caregiver willingness to repeat the test	63/63	100%
Child willingness to repeat the test	63/63	100%
Wong Baker FACES® scale during the procedure	63/63	100%
Wong Baker FACES® scale after the procedure	63/63	100%

### Feasibility

Of the 148 children, 50 (34%) successfully swallowed the capsule. More children in the 11–14 year age group were able to swallow the capsule as compared to children in the 4–10 year age group (83% vs 22% respectively, *p* < 0.0001). Among the 72 children that could not produce a spontaneous sputum sample, 13/66 children in the 4–10 group (20%) and 6/6 children in 11–14 group (100%) successfully completed the string test.

### Acceptability among children and guardians

Table 3 shows that 39% of children aged 4 to 10 years old were willing to repeat the string test, while 21% were willing to repeat gastric aspiration (*p* = 0.017). In contrast, guardians of younger children were more often willing to have their child repeat gastric aspiration as compared to the string test (96% vs 81%, *p* = 0.005). Children 11 to 14 years old and their guardians were more likely to report willingness to repeat the gastric aspirate procedure as compared to the string test (86% vs. 58% in children; *p* = 0.182, 100% vs. 83% in guardians). While more caregivers reported comfort during the string test than during the gastric aspirate procedure, nearly all caregivers reported comfort with both the string test and gastric aspiration after they were completed (Table 3).

**Table 3:** Acceptability of string test and gastric aspirate procedures among children and their guardians

	4–10 years old					11–14 years old				
	String test		Gastric aspirate		*p*-value	String test		Gastric aspirate		*p*-value
	n/N	%	n/N	%		n/N	%	n/N	%	
Guardian reported comfort with the procedure:										
*During*	24/25	96	35/56	63	0.001	21/21	100	4/7	57	**
*After*	83/88	94	53/56	95	0.925	23/24	96	7/7	100	**
Willing to repeat the procedure:										
*Child*	34/88	39	12/56	21	0.017	15/26	58	6/7	86	0.182
*Parent*	71/88	81	54/56	96	0.005	20/24	83	7/7	100	**

**Clustered p-value could not be calculated due to a cell count of zero

### Tolerability

Figure 1 shows that nearly all younger children (95%) reported at least some pain during the gastric aspirate procedure and 39% reported the highest level (‘hurts worst’). In contrast, during the string test, only 52% reported pain and 4% reported the highest level. All older children (100%) reported at least some pain during the gastric aspiration whereas only 41% reported any pain during the string test.

### Diagnostic effectiveness

Table 4 shows culture positivity by sample type by age group in children with a successful string test. Among children with both a string and gastric aspirate sample, culture positivity was 5% for both sample types. Among the 34 children with both a string and sputum sample (either spontaneously expectorated or induced), 9 had a positive sputum culture, of which six were positive by string culture. All six of the positive cultures from string were among children 11–14 years old. Among the 21 children with both a string and gastric aspirate sample, one children had a positive gastric aspirate culture and a positive string culture. Every child with a positive string culture also had a positive culture by sputum or gastric aspirate.

**Table 4:** *Mycobacterium tuberculosis* culture positivity by sample type among 50 children with a successful string test

Sample type	4–10 years old		11–14 years old		Total	
	*N = 26*		*N = 24*		*N = 50*	
	n/N	%	n/N	%	n/N	%
String vs. Gastric Aspirate						
String test	0/ 14	0	1/7	14	1/21	5
Gastric aspirate	0/14	0	1/7	14	1/21*	5
String vs. Sputum						
String test	0/ 15	0	6/19	32	6/34	18
Spontaneous sputum	1/13	8	7/18*	41	8/31	26
Induced sputum	0/2	0	1/2*	50	1/4*	25

*Children with multiple respiratory sample types (spontaneous sputum, induced sputum, gastric aspirate) are included in the denominator of each sample type; therefore, the denominator is greater than the number of children. In the 11–14 age group: one child provided one spontaneous and one induced sputum sample, both culture negative. Another child in this group provided one gastric aspirate and one induced sputum; both culture positive

## Discussion

This study demonstrated that the string test, although generally tolerable and accepted by children and their caregivers, was not reliably feasible, especially among younger children. This is the group least likely to be able to produce a spontaneous sputum sample and therefore, in greatest need of an alternative sample type. There were no positive string cultures in this group. We did not observe increased bacteriological detection when using the string test as compared to conventional sample types. Among children who were able to produce a sputum sample (either spontaneously or through induction), we found that culture positivity for the string test was lower than sputum (6 of 9 children with positive sputum cultures, had positive string cultures). The single gastric aspirate sample in our study that was positive by culture was also positive by string.

Most children in the 4–10 year-old age group (78%) were not able to swallow the capsule. In contrast, older children were generally able to swallow the capsule and successfully complete the test. String test success rates were higher (> 80%) in other studies with a similar median age [4, 16, 17]; though these studies also found the string test to be least successful in young children. Had gastric aspirate and sputum induction not been available in our setting, the string test would have resulted in a sample for culturing in 13 (20%) children 4–10 and 6 (100%) children 11–14 who would not have otherwise had one because they could not produce a spontaneous sputum sample.

Our capsule sizes for children < 10 years were the same size or smaller than the pediatric Entero-Test (HDC Corporation, San Jose, CA), which was used in previous studies [4, 17] or adapted from Entero-test [18]. Smaller capsules and additional interventions to facilitate pill swallowing [25] could improve string test feasibility. One potential explanation for differential string test success across settings relates to pill taking familiarity. For example, one third of children that participated in the study conducted in Uganda by Nansumba, et al. [4] were living with HIV, and therefore may have been more accustomed to swallowing pills.

For the diagnosis of pediatric TB in primary health centers, the string test may have advantages over gastric aspiration, which requires more supplies, experienced, well-trained personnel and an adequate environment in which to perform the procedure. Both methods require overnight fasting. Notably, older children and guardians of children in both age groups were more likely to report willingness to repeat gastric aspiration than to repeat the string test. Study nurses reported that many guardians praised the brevity of gastric aspiration as compared to the long duration of the string test, which could be disruptive to daily activities. While gastric aspirate takes an average of 20 min and is completed in one attempt, the string test requires up to 4 h of intragastric downtime. Although one study in adults suggested that intragastric downtime could be reduced to one hour without a loss in diagnostic yield, it is unknown to what extent these findings apply to children, who are more likely to have paucibacillary disease [26]. Though longer, most children reported that the string test was relatively painless, as compared to the gastric aspirate.

We compared the diagnostic yield from a single string sample to the yield from up two sputum and/or gastric aspirate samples. An additional string sample may have improved detection relative to sputum; however, given that the string test would be most valuable in children unable to produce sputum, we do not perceive this as a major study limitation. A second limitation is the small sample number of children with culture-confirmed TB, which precluded us from formally testing the diagnostic yield of the string sample relative to conventional respiratory samples.

## Conclusions

Our findings reinforce those from a small body of literature examining the string test as a diagnostic specimen for children with suspected TB. The string test is a well-tolerated and acceptable alternative sample type for diagnosing TB in children among those who are able to swallow the capsule. In contexts where gastric aspirate is not available, it may be an acceptable substitute; however, the sample and case detection yield may be small to moderate.

## Abbreviations

GA: Gastric aspirate
HIV: Human Immunodeficiency Virus
MoH: Ministry of Health
rpm: Revolutions per minute
TB: Tuberculosis
TST: Tuberculin Skin Test
WHO: World Health Organization
